# The development of a three-frame timeline for clinical concepts extracted from discharge summaries

**DOI:** 10.1186/1471-2105-13-S12-A14

**Published:** 2012-07-31

**Authors:** Christopher Burton, Sujin Kim

**Affiliations:** 1 Department of Computer Science, Eastern Kentucky University, Richmond, KY 40475, USA; 2 Division of Biomedical Informatics, Department of Biostatistics, University of Kentucky, Lexington, KY 40506, USA

## Background

Through the functional presentation of information, natural language processing (NLP) of electronic medical records (EMR) improves efficiency in the clinical research and patient care domains. Discharge summary NLP, when utilized to associate clinical concepts with temporal references, conveys the order of events related to an admission.

## Materials and methods

Utilizing Knowtator, a schema was designed to relate clinical concepts within discharge summary sections, including disease states, procedures, lab results, and medications, to a timeline. The timeline was defined by three frames: Pre-Admission, Hospital Stay, and Post-Discharge. Fifty-five discharge summaries from the i2b2 NLP Challenge were randomly selected for review. Clinical concepts were manually extracted, categorized within the appropriate frame, and compared to the MetaMAP and YTEx systems to establish a gold standard. It was observed that concepts in the same section correlated to the same frame in the timeline. Additionally, the frame was determined through the classification of section titles. The same method could be further refined to associate clinical concepts with more specific date and time markers, leading to a comprehensive view of the medical timeline.

